# Cancer prevalence, incidence, and mortality rates in Afghanistan in 2020: A review study

**DOI:** 10.1002/cnr2.1873

**Published:** 2023-08-13

**Authors:** Nasar Ahmad Shayan, Ali Rahimi, Hilal Özcebe

**Affiliations:** ^1^ Department of Epidemiology and Biostatistics, Schulich School of Medicine and Dentistry Western University London Ontario Canada; ^2^ Scientific Research Center Jami University Herat Afghanistan; ^3^ Department of Public Health, Faculty of Medicine Hacettepe University Ankara Turkey

**Keywords:** Afghanistan, cancer, incidence, mortality, prevalence, risk factors

## Abstract

**Background:**

Afghanistan is in an epidemiological transition, as cancer is the second leading cause of mortality due to non‐communicable diseases. This study is the first to provide a comprehensive perspective on the overall cancer situation in Afghanistan by discussing the top five most common cancers, their incidence variations, risk factors, and preventive measures. The limited number of cancer studies conducted in Afghanistan highlights the importance of the present review.

**Recent Findings:**

This article provides an overview of cancer burden in Afghanistan in 2020. It utilizes IARC‐generated GLOBOCAN 2020 data for one, three, and five‐year prevalence rates, the estimated number of new cancer cases, and mortality rates by age group in Afghanistan. According to GLOBOCAN, the top five common cancers in both sexes in Afghanistan were breast (*n* = 3173, 14.3%), stomach (*n* = 2913, 7.8%), lung (*n* = 1470, 6.6%), cervix uteri (*n* = 1200, 5.4%), and colorectum (*n* = 1084, 4.9%).

**Conclusion:**

This study provides a brief overview of the general cancer situation in Afghanistan, and a more in‐depth analysis of the five common cancers identified. Effective therapies, awareness, and prevention initiatives targeting lifestyle, immunization, early diagnosis, and environmental risk factors are essential for addressing the impact of population growth and aging on cancer incidence in Afghanistan. Further research and extensive studies are needed to better understand cancer burden in the country.

## 1 INTRODUCTION

Cancer is a primary cause of mortality and the main impediment to improving life expectancy worldwide.^1^ In 2020, there were approximately 18 million new cancer diagnoses and 10 million cancer‐related deaths worldwide. According to the World Health Organization (WHO) predictions for 2019, cancer is the primary or second major cause of death before 70 years old in 112 of 183 countries and ranks third or fourth in another 23.^2^ Cancer is gaining significance as a primary cause of death partially due to substantial declines in stroke and coronary heart disease mortality rates relative to cancer in many nations.^1^ Consequently, the global burden of cancer incidence and death has rapidly increased; this includes both population aging and growth, as well as changes in the prevalence and distribution of cancer risk factors, some of which are related to socioeconomic development.^3^

Afghanistan is a landlocked and mountainous country with a population of 38 million people located within South Asia and Central Asia, bordering China, Pakistan, Iran, Turkmenistan, Uzbekistan, and Tajikistan. According to the WHO, life expectancy at birth in Afghanistan is 60 years for men and 61 years for women. Afghanistan is in an epidemiologic transition and faces a double disease burden. Cancer is the second leading cause of mortality due to non‐communicable diseases in Afghanistan. According to the most recent WHO Afghanistan country profile report, 19 450 cancer cases and 14 746 cancer‐related deaths were reported in 2018. According to this report, breast, stomach, lip/oral cavity, esophagus, and lung cancers had the highest cancer incidence, and so did cancer‐related deaths.^4^ As very few studies on the epidemiology and risk factors of cancer have been conducted in Afghanistan, WHO reports are the only reliable source for this nation.^5^, ^6^, ^7^, ^8^

The Cancer Surveillance Branch (CSU) at the International Agency for Research on Cancer (IARC) sub agency of the World Health Organization (WHO) is responsible for providing regular worldwide estimates of cancer burden. GLOBOCAN 2020 revises previously reported cancer incidence and death figures for 2018.^9^, ^10^ The basic estimation units are nations, with aggregated findings globally and in 20 geographical regions established by the United Nations (UN).^11^ Estimations were calculated for 38 cancer sites, including other and nonspecific cancers, by sex and 18 predefined age groups. Estimation methodologies and determination of uncertainty intervals continue to rely on the best available data on cancer incidence and death at the national level. The Global Cancer Observatory (GCO) provides interactive tabulation and graphical visualization of the GLOBOCAN data collection for 185 nations and geographical regions by sex. There is a more comprehensive description of regional variability across 20 global areas.^12^

To date, no study has published the age‐standardized and cancer‐specific incidence and death rates in men and women in Afghanistan. Therefore, this article presents an overview of the cancer burden in Afghanistan, including the estimated number of new cancer cases and mortalities by age group in 2020. It also provides 1‐, 3‐, and 5‐year cancer prevalence in Afghanistan and discusses cancer's scale and profile, risk factors for a variety of top five common cancers, and preventative approaches that potentially can decrease future cancer burdens.

## 2 METHODS

The GLOBOCAN 2020 database was used as the primary source of information to obtain data presented in the tables. Data on cancer incidence, death, and prevalence were collected from population‐based cancer registries (PBCR) in Afghanistan. PBCRs provide data on cancer incidence and are crucial for developing and evaluating cancer control programs. Cancer mortality data was collected from the WHO.^10^, ^12^ The estimates presented in this study do not consider the influence of the COVID‐19 pandemic caused by the SARS‐CoV‐2 virus and recent political change in Afghanistan because they are based on extrapolated data gathered from these events.

The authors used cancer‐specific estimates available in the GCO for 36 cancer types based on codes from the International Statistical Classification of Diseases and Related Health Problems, 10th Revision (ICD‐10). Nonmelanoma skin cancer (NMSC) was excluded from the study because of difficulty in tracking it in cancer registries. The study provides estimates for Afghanistan based on sex and 9 age groups: 0–14, 14–39, 40–44, 45–49, 50–54, 55–59, 60–64, 65–69, and 75 years and above.

The figures were presented as age‐standardized rates (ASR) per 100 000 person‐years, based on Segi's World Standard Population, as proposed by Segi and adjusted by Doll and Cook.^13^, ^14^ The cumulative risk of developing or experiencing cancer‐related death before age 70 was also estimated and given as a percentage.

The authors did not perform any calculations but used the data provided by GLOBOCAN to create tables that presented the incidence, mortality, and prevalence of cancer in Afghanistan by gender and age groups. The tables were created by combining and revising the data provided in GLOBOCAN to fit the authors' needs. The use of GLOBOCAN data is free of charge for non‐commercial purposes.

The data for this study was obtained from GLOBOCAN 2020 database, and the authors used the available cancer‐specific estimates to create tables presenting the incidence, mortality, and prevalence of cancer in Afghanistan by sex and age group. The figures were presented as age‐standardized rates (ASR), and the cumulative risk of developing or experiencing cancer‐related death before age 70 was estimated and given as a percentage.

In the discussion section, we conducted a literature review to examine the global, regional, and Afghanistan‐specific epidemiology, possible risk factors, and prevention and cure measures for the top five cancers in Afghanistan. Our review included peer‐reviewed articles, policy documents, reports, and guidelines. To gather the necessary information, we employed a search strategy across multiple databases, including PubMed, Scopus, and Google Scholar. We used a combination of keywords, such as “cancer,” “epidemiology,” “incidence,” “mortality,” “prevalence,” and “Afghanistan” to ensure a broad coverage of relevant literature. We utilized BOOLEAN operators and MeSH terms to refine our search and increase its specificity (Table 1). Additionally, we employed a snowballing technique to identify additional relevant articles. This involved analyzing the reference lists of the selected papers and including those that met our inclusion criteria for further analysis. Our inclusion criteria focused on selected articles in English that discussed key concepts including cancer, risk factors, challenges, policy recommendations, and Afghanistan, Asia, and World cancer statistics in 2020. This allowed us to incorporate a range of perspectives and insights into the topic. Furthermore, we considered online available documents related to Afghanistan, including the Ministry of Public Health of Afghanistan and other reputable sources. Although rare, the findings from these sources provided important insights into the burden of cancer in Afghanistan and highlighted the need for better prevention and control measures. Overall, this discussion section sheds light on the current state of cancer in Afghanistan and provides recommendations for future research and interventions to improve cancer outcomes in the country.

**TABLE 1:** Search strategy terms in selected databases.

Database	Query	Articles
Scopus	TITLE‐ABS‐KEY (cancer) AND TITLE‐ABS‐KEY (Afghanistan) AND TITLE‐ABS‐KEY (breast OR stomach OR gastric OR cervical OR lung OR pulmonary OR colorectum OR colon OR rectum OR anus) AND TITLE‐ABS‐KEY (epidemiology OR incidence OR prevalence OR mortality) AND (LIMIT‐TO [LANGUAGE, “English”])	42
PubMed	((Afghanistan[Title/Abstract]) OR (Afghanistan[MeSH Terms])) AND ((cancer[Title/Abstract]) OR (cancer[MeSH Terms])) AND (((breast[Title/Abstract]) OR (stomach[Title/Abstract]) OR (gastric[Title/Abstract]) OR (cervical[Title/Abstract]) OR (lung[Title/Abstract]) OR (pulmonary[Title/Abstract]) OR (colorectum[Title/Abstract]) OR (colon[Title/Abstract]) OR (rectum[Title/Abstract]) OR (anus[Title/Abstract])) OR ((breast[MeSH Terms]) OR (stomach[MeSH Terms]) OR (gastric[MeSH Terms]) OR (cervical[MeSH Terms]) OR (lung[MeSH Terms]) OR (pulmonary[MeSH Terms]) OR (colon[MeSH Terms]) OR (rectum[MeSH Terms]) OR (anus[MeSH Terms]))) AND (((epidemiology[Title/Abstract]) OR (incidence[Title/Abstract]) OR (prevalence[Title/Abstract]) OR (mortality[Title/Abstract])) OR ((epidemiology[MeSH Terms]) OR (incidence[MeSH Terms]) OR (prevalence[MeSH Terms]) OR (mortality[MeSH Terms])))	21
Google Scholar	“Afghanistan” “cancer” (10 pages reviewed)	100
	Afghanistan “cancer” epidemiology prevalence incidence mortality breast stomach gastric cervical lung pulmonary colorectum colon rectum anus	104

## 3 RESULTS

### 3.1 Prevalence

In 2020, the most common cancers among Afghanistan's male population were stomach, lung, lip/oral cavity, leukemia, and colorectal, while breast, cervix uteri, stomach, corpus uteri, and ovarian cancers were the most common among women (Table 2).

**TABLE 2:** Incidence and estimated 1, 3, and 5‐year prevalent cancer cases in Afghanistan population, 2020.

		Male				Female			
Cancer	ICD	Incidence	1‐year (prop.)	3‐year (prop.)	5‐year (prop.)	Incidence	1‐year (prop.)	3‐year (prop.)	5‐year (prop.)
**Gastrointestinal**									
Stomach	C16	1342	702 (3.5)	1364 (6.8)	1761 (8.8)	807	426 (2.2)	867 (4.6)	1152 (6.1)
Lip, oral cavity	C00‐06	618	295 (1.5)	725 (3.6)	1042 (5.2)	295	147 (0.78)	368 (1.9)	536 (2.8)
Colorectum	C18‐21	594	276 (1.4)	681 (3.4)	944 (4.7)	490	234 (1.2)	580 (3.1)	835 (4.4)
Esophagus	C15	569	295 (1.5)	506 (2.5)	610 (3.1)	451	231 (1.2)	415 (2.2)	506 (2.7)
Liver	C22	553	280 (1.4)	534 (2.7)	680 (3.4)	403	215 (1.1)	401 (2.1)	502 (2.6)
Salivary glands	C07‐08	34	16 (0.08)	46 (0.23)	68 (0.34)	27	13 (0.07)	39 (0.21)	63 (0.33)
Pancreas	C25	222	109 (0.55)	182 (0.91)	217 (1.1)	143	75 (0.4)	127 (0.67)	155 (0.82)
Oropharynx	C09‐10	104	50 (0.25)	122 (0.61)	176 (0.88)	47	21 (0.11)	53 (0.28)	77 (0.41)
Hypopharynx	C12‐13	98	37 (0.19)	78 (0.39)	99 (0.5)	54	24 (0.13)	50 (0.26)	66 (0.35)
**Respiratory**									
Lung	C33‐34	1070	536 (2.7)	941 (4.7)	1151 (5.8)	400	228 (1.2)	441 (2.3)	562 (3)
Larynx	C32	204	97 (0.49)	248 (1.2)	358 (1.8)	47	21 (0.11)	59 (0.31)	87 (0.46)
Nasopharynx	C11	85	42 (0.21)	112 (0.56)	170 (0.85)	36	16 (0.08)	45 (0.24)	67 (0.35)
Mesothelioma	C45	11	6 (0.03)	14 (0.07)	18 (0.09)	3	3 (0.02)	3 (0.02)	4 (0.02)
**Reproductive system**									
Prostate	C61	450	209 (1)	493 (2.5)	655 (3.3)				
Testis	C62	182	95 (0.48)	266 (1.3)	420 (2.1)				
Penis	C60	11	5 (0.03)	13 (0.07)	22 (0.11)				
Breast	C50					3173	1597 (8.4)	4115 (21.7)	5930 (31.3)
Cervix uteri	C53					1200	581 (3.1)	1446 (7.6)	2089 (11)
Corpus uteri	C54					519	257 (1.4)	659 (3.5)	964 (5.1)
Ovary	C56					495	240 (1.3)	627 (3.3)	918 (4.8)
Vulva	C51					39	19 (0.1)	51 (0.27)	79 (0.42)
Vagina	C52					20	6 (0.03)	24 (0.13)	37 (0.2)
**Urologic**									
Bladder	C67	377	183 (0.92)	468 (2.3)	681 (3.4)	78	36 (0.19)	89 (0.47)	125 (0.66)
Kidney	C64‐65	386	181 (0.91)	458 (2.3)	665 (3.3)	222	109 (0.58)	293 (1.5)	457 (2.4)
Gallbladder	C23	49	30 (0.15)	53 (0.27)	67 (0.34)	95	56 (0.3)	99 (0.52)	120 (0.63)
**Blood and lymphoid tissues**									
Leukemia	C91‐95	606	300 (1.5)	836 (4.2)	1317 (6.6)	472	229 (1.2)	642 (3.4)	1032 (5.4)
Non‐Hodgkin lymphoma	C82‐86, C96	348	167 (0.84)	453 (2.3)	684 (3.4)	236	114 (0.6)	309 (1.6)	464 (2.4)
Kaposi sarcoma	C46	18	8 (0.04)	22 (0.11)	35 (0.18)	10	4 (0.02)	12 (0.06)	
Hodgkin lymphoma	C81	138	67 (0.34)	185 (0.93)	280 (1.4)	99	50 (0.26)	141 (0.74)	218 (1.2)
Multiple myeloma	C88 + C90	69	31 (0.16)	77 (0.39)	104 (0.52)	59	26 (0.14)	63 (0.33)	86 (0.45)
**Nervous**									
Brain, central nervous system	C70‐72	588	280 (1.4)	722 (3.6)	1079 (5.4)	427	208 (1.1)	563 (3)	856 (4.5)
**Others**									
Thyroid	C73	65	33 (0.17)	86 (0.43)	132 (0.66)	195	99 (0.52)	272 (1.4)	418 (2.2)
Melanoma of skin	C43	65	33 (0.17)	83 (0.42)	123 (0.62)	47	23 (0.12)	62 (0.33)	93 (0.49)
**All cancers excl. non‐melanoma skin cancer**	C00‐97/C44	10 246	5101 (25.5)	11 533 (57.7)	16 101 (80.6)	12 003	6094 (32.2)	14 816 (78.2)	21 272 (112.2)

*Note*: The incidence, prevalence proportions (prop.), crude rates, and ASR were reported per 100 000 people.

### 3.2 Incidence

In 2020, an estimated 10 246 new cancer cases (approximately 28 newly diagnosed cancer cases per day) emerged in the Afghan male population. The top five commonly diagnosed cancers in males are respectively stomach (*n* = 1342, 13.10%, crude = 6.7, ASR = 16.2, CR = 4.21); lung (*n* = 1070, 10.44%, crude = 5.4, ASR = 12.3, CR = 2.71); lip and oral cavity (*n* = 618, 6.03%, crude = 3.1, ASR = 6.4, CR = 1.44); leukemia (*n* = 606, 5.91%, crude = 3, ASR = 3.5, CR = 0.48); and colorectum (*n* = 594, 5.80%, crude = 3, ASR = 6.3) (Table 3). From all diagnosed cancer cases in this population, 7.05% (*n* = 722) were diagnosed in children aged 0–14 years, 62.68% (*n* = 5869) in adults aged 15–64 years, and 30.28% (*n* = 2483) in the elderly population aged more than 65 years.

**TABLE 3:** Estimated number of new cancer cases by age in Afghanistan male population, 2020.

Cancer	Total	0–14	15–39	40–44	45–49	50–54	55–59	60–64	65–69	75+	Crude Rate	ASR (World)	Cum. risk
**Stomach**	1342 (13.10%)	2	70	38	82	139	202	230	220	359	6.7	16.2	4.21
**Lung**	1070 (10.44%)	6	48	55	95	133	168	181	163	221	5.4	12.3	2.71
**Lip, oral cavity**	618 (6.03%)	6	107	56	63	71	75	73	63	104	3.1	6.4	1.44
**Leukemia**	606 (5.91%)	244	208	27	25	23	22	18	15	24	3	3.5	0.48
**Colorectum**	594 (5.80%)		105	40	49	58	72	80	76	114	3	6.3	–
Brain, central nervous system	588 (5.74%)	92	205	54	54	50	45	35	25	28	2.9	4.3	0.57
Esophagus	569 (5.55%)		24	17	35	53	72	85	88	195	2.8	7.3	2.44
Liver	553 (5.40%)	18	60	25	41	56	71	79	76	127	2.8	6.2	1.58
Prostate	450 (4.39%)		8	5	6	10	40	81	112	188	2.3	6.1	1.85
Kidney	386 (3.77%)	41	55	34	41	46	46	41	33	49	1.9	3.7	0.73
Bladder	377 (3.68%)		31	16	26	37	51	62	62	92	1.9	4.4	1.07
Non‐Hodgkin lymphoma	348 (3.40%)	51	114	27	27	28	27	25	21	28	1.7	2.7	0.44
Pancreas	222 (2.17%)	1	20	10	15	23	30	35	35	53	1.1	2.6	0.65
Larynx	204 (1.99%)		11	12	19	26	34	35	29	38	1.0	2.3	0.47
Testis	182 (1.78%)	19	117	17	11	7	5	3	2	1	0.91	1.0	0.09
Hodgkin lymphoma	138 (1.35%)	40	60	8	6	6	6	4	4	4	0.69	0.79	0.08
Oropharynx	104 (1.02%)	1	7	4	8	11	14	17	15	27	0.52	1.2	0.26
Hypopharynx	98 (0.96%)		6	4	3	12	17	20	16	20	0.49	1.2	0.27
Nasopharynx	85 (0.83%)	8	31	7	7	6	7	7	6	6	0.43	0.66	0.1
Multiple myeloma	69 (0.67%)		27	5	3	5	5	6	7	11	0.35	0.67	0.19
Thyroid	65 (0.63%)	2	17	5	9	10	8	6	5	3	0.33	0.56	0.07
Melanoma of skin	65 (0.63%)	1	17	7	3	3	4	6	9	15	0.33	0.67	0.19
Gallbladder	49 (0.48%)		5	1	4	5	7	8	7	12	0.25	0.58	0.16
Salivary glands	34 (0.33%)		9	2	2	3	4	4	4	6	0.17	0.33	0.06
Kaposi sarcoma	18 (0.18%)		5	5			1	2	3	2	0.09	0.16	0.03
Penis	11 (0.11%)			1	1	2	2	1	1	3	0.06	0.12	0.02
Mesothelioma	11 (0.11%)		4	1	2		2	1	1		0.06	0.09	0.01
**All cancers excluding non‐melanoma skin cancer**	**10 246 (100.00%)**	722 (7.05%)	1676 (16.36%)	585 (5.71%)	757 (7.39%)	954 (9.31%)	1175 (11.47%)	1275 (12.44%)	1208 (11.79%)	1894 (18.49%)	51.3	104.5	21.45

*Note*: The incidence, prevalence proportions (prop.), crude rates, and ASR were reported per 100 000 people.

On the other hand, the estimated number of new cancer cases among females in 2020 is 12 003 (approximately 33 new cancer cases diagnosed per day). The top five commonly diagnosed cancers in females are respectively breast (*n* = 3173, 26.44%, crude = 16.7, ASR = 28.9, CR = 4.3), cervix uteri (*n* = 1200, 10.00%, crude = 6.3, ASR = 10.4, CR = 1.4), stomach (*n* = 807, 6.72%, crude = 4.3, ASR = 8.9, CR = 2.31), corpus uteri (*n* = 519, 4.32%, crude = 2.7, ASR = 4.9, CR = 0.69), and ovary (*n* = 495, 4.12%, crude = 2.6, ASR = 4.1, CR = 0.53) (Table 4). From all newly diagnosed cases, 6.08% (*n* = 623) were diagnosed in children aged 0–14 years, 86.54% (*n* = 8379) in adults aged 15–64 years, and 24.53% (*n* = 2072) in the elderly aged above 65 years.

**TABLE 4:** Estimated number of new cancer cases by age in Afghanistan female population, 2020.

Cancer	Total	0–14	15–39	40–44	45–49	50–54	55–59	60–64	65–69	70+	Crude Rate	ASR (World)	Cum. risk
**Breast**	3173 (26.44%)		871	386	393	379	340	287	221	296	16.7	28.9	4.3
**Cervix uteri**	1200 (10.00%)	1	394	169	161	142	114	86	59	74	6.3	10.4	1.4
**Stomach**	807 (6.72%)	2	67	43	58	77	98	115	117	230	4.3	8.9	2.31
**Corpus uteri**	519 (4.32%)		102	68	74	73	66	55	40	41	2.7	4.9	0.69
**Ovary**	495 (4.12%)	11	180	58	58	54	45	35	25	29	2.6	4.1	0.53
Colorectum	490 (4.08%)		70	33	35	43	54	65	71	119	2.6	5.1	‐
Leukemia	472 (3.93%)	175	160	25	23	22	20	16	13	18	2.5	2.9	0.33
Esophagus	451 (3.76%)	1	41	22	32	41	52	63	67	132	2.4	5.0	1.29
Brain, central nervous system	427 (3.56%)	78	170	34	32	29	25	21	16	22	2.3	3.0	0.38
Lung	400 (3.33%)	3	67	27	34	39	45	50	49	86	2.1	4.1	0.9
Liver	403 (3.36%)	15	43	13	21	30	41	51	55	134	2.1	4.4	1.35
Lip, oral cavity	295 (2.46%)	4	53	29	33	35	35	33	28	45	1.6	2.9	0.53
Non‐Hodgkin lymphoma	236 (1.97%)	31	90	20	19	18	16	13	11	18	1.2	1.8	0.27
Kidney	222 (1.85%)	48	47	17	19	20	19	17	14	21	1.2	1.8	0.28
Thyroid	195 (1.62%)	3	64	19	22	23	21	17	12	14	1.0	1.7	0.22
Pancreas	143 (1.19%)		17	7	9	13	17	21	20	39	0.75	1.6	0.4
Hodgkin lymphoma	99 (0.82%)	14	63	4	2	5	4	3	3	1	0.52	0.57	0.05
Gallbladder	95 (0.79%)		5	6	9	11	12	15	13	24	0.5	1.1	0.26
Bladder	78 (0.65%)	4	14	7	8	8	7	8	8	14	0.41	0.76	0.16
Multiple myeloma	59 (0.49%)		15	7	3	6	6	5	5	12	0.31	0.57	0.13
Hypopharynx	54 (0.45%)		10	5	7	8	7	6	5	6	0.28	0.52	0.08
Oropharynx	47 (0.39%)	2	5	5	8	5	5	4	6	7	0.25	0.46	0.08
Melanoma of skin	47 (0.39%)		10	4	4	4	5	4	3	13	0.25	0.47	0.12
Larynx	47 (0.39%)		7	4	9	8	6	4	3	6	0.25	0.47	0.1
Vulva	39 (0.32%)		3	2	4	4	4	7	6	9	0.21	0.42	0.08
Nasopharynx	36 (0.30%)	1	17	3	3	2	2	3	3	2	0.19	0.28	0.04
Salivary glands	27 (0.22%)		8	2	2	1	2	4	3	5	0.14	0.27	0.07
Vagina	20 (0.17%)		2	1	1	3	3	3	3	4	0.11	0.21	0.03
Kaposi sarcoma	10 (0.08%)		6				2	1		1	0.05	0.07	0.01
Mesothelioma	3 (0.02%)						1	1	1		0.02	0.04	0.01
**All cancers excl. non‐melanoma skin cancer**	12 003 (100.00%)	623 (6.08%)	3031 (29.58%)	1127 (11.00%)	1196 (11.67%)	1218 (11.89%)	1184 (11.56%)	1111 (10.84%)	961 (9.38%)	1552 (15.15%)	63.3	108.7	17.7

*Note*: The incidence, prevalence proportions (prop.), crude rates, and ASR were reported per 100 000 people.

### 3.3 Mortality

In 2020, the Afghan male population faced an estimated 7891 cancer deaths (about 22 cancer deaths per day). The top five cancers responsible for male death are stomach (*n* = 1228, 15.56%, crude = 6.1, ASR = 14.9, CR = 3.85); lung (*n* = 993, 12.58%, crude = 5, ASR = 11.5, CR = 2.52); esophagus (*n* = 547, 6.93%, crude = 2.7, ASR = 6.9, CR = 2.24); Liver (*n* = 583, 6.88%, crude = 2.7, ASR = 6, CR = 1.36); and brain and central nervous system (*n* = 511, 6.48%, crude = 2.6, ASR = 4, CR = 0.56) (Table 5). From all male cancer mortalities, 3.99% (*n* = 409) were children aged 0–14 years, 47.65% (*n* = 4193) were adults aged 15–64 years, and 25.38% (*n* = 2113) were elderly aged above 65 years.

**TABLE 5:** Estimated number of cancer death cases by age in Afghanistan male population, 2020.

Cancer	Total	0–14	15–39	40–44	45–49	50–54	55–59	60–64	65–69	70+	Crude Rate	ASR (World)	Cum. risk
**Stomach**	1228 (15.56%)	2	66	25	68	129	189	216	206	327	6.1	14.9	3.85
**Lung**	993 (12.58%)	4	40	48	90	130	165	170	147	199	5.0	11.5	2.52
**Esophagus**	547 (6.93%)		20	16	35	55	75	85	84	177	2.7	6.9	2.24
**Liver**	543 (6.88%)	10	61	26	42	58	74	82	78	112	2.7	6	1.36
**Brain, central nervous system**	511 (6.48%)	59	162	49	51	50	46	37	27	30	2.6	4	0.56
Lip, oral cavity	433 (5.49%)	3	68	34	42	51	59	58	48	70	2.2	4.5	0.97
Leukemia	434 (5.50%)	136	162	24	22	20	19	15	13	23	2.2	2.7	0.42
Colorectum	382 (4.84%)		58	23	25	38	50	53	51	84	1.9	4.2	‐
Prostate	272 (3.45%)		6	3	2	5	24	48	63	121	1.4	3.7	1.26
Non‐Hodgkin lymphoma	259 (3.28%)	27	81	19	21	23	23	21	18	26	1.3	2.1	0.37
Kidney	268 (3.40%)	22	21	15	23	32	39	38	32	46	1.3	2.8	0.6
Bladder	234 (2.97%)		10	6	10	20	31	40	41	76	1.2	2.9	0.8
Pancreas	215 (2.72%)	1	20	9	15	22	30	35	34	49	1.1	2.5	0.58
Larynx	155 (1.96%)		8	4	9	17	25	31	28	33	0.78	1.8	0.4
Oropharynx	68 (0.86%)	1	4	2	6	7	10	12	10	16	0.34	0.77	0.16
Nasopharynx	63 (0.80%)	5	22	7	6	4	5	6	4	4	0.32	0.5	0.07
Hodgkin lymphoma	63 (0.80%)	20	20	3	3	4	4	3	2	4	0.32	0.41	0.05
Testis	59 (0.75%)	14	28	4	3	3	3	2	1	1	0.3	0.35	0.03
Multiple myeloma	58 (0.74%)		23	4	2	4	4	6	7	8	0.29	0.55	0.13
Hypopharynx	44 (0.56%)		4	2	1	3	5	8	9	12	0.22	0.52	0.12
Melanoma of skin	37 (0.47%)	1	8	4	1	2	3	5	5	8	0.19	0.38	0.09
Gallbladder	38 (0.48%)		4	1	3	4	5	6	5	10	0.19	0.45	0.14
Thyroid	28 (0.35%)	1	4	1	3	4	5	5	3	2	0.14	0.28	0.04
Salivary glands	22 (0.28%)		4	1	1	2	3	3	3	5	0.11	0.23	0.04
Kaposi sarcoma	13 (0.16%)		2	3			1	2	3	2	0.07	0.13	0.02
Mesothelioma	10 (0.13%)		3	1	2		2	1	1		0.05	0.08	0.01
Penis	5 (0.06%)					1	1	1		2	0.03	0.06	0.01
**All cancers excluding non‐melanoma skin cancer**	7891 (100.00%)	409 (3.99%)	1018 (9.94%)	394 (3.85%)	569 (5.55%)	789 (7.70%)	1014 (9.90%)	1098 (10.72%)	1015 (9.91%)	1585 (15.47%)	39.5	84.3	18.06

*Note*: The incidence, prevalence proportions (prop.), crude rates, and ASR were reported per 100 000 people.

In comparison, the total number of female deaths was estimated to be 7905 (approximately 22 cancer deaths per day). The top five cancers causing death in females are breast (*n* = 1783, 22.60%, crude = 9.4, ASR = 17.9, CR = 3.2), cervix uteri (*n* = 823, 10.43%, crude = 4.3, ASR = 7.6, CR = 1.11), stomach (*n* = 690, 8.74%, crude = 3.6, ASR = 7.8, CR = 2.03), esophagus (*n* = 436, 5.53%, crude = 2.3, ASR = 4.8, CR = 1.21), and brain and central nervous system (*n* = 386, 4.89%, crude = 2, ASR = 2.9, CR = 0.39) (Table 6). Of all female cancer mortalities, 3.32% (*n* = 340) occurred in children aged 0–14 years, 53.01% (*n* = 4869) occurred in adults aged 15–64 years, and 20.83% (*n* = 1711) occurred in the elderly population aged above 65 years.

**TABLE 6:** Estimated number of cancer death cases by age in Afghanistan's female population, 2020.

Cancer	Total	0–14	15–39	40–44	45–49	50–54	55–59	60–64	65–69	70+	Crude Rate	ASR (World)	Cum. risk
**Breast**	1783 (22.60%)		281	174	203	225	229	216	183	272	9.4	17.9	3.2
**Cervix uteri**	823 (10.43%)	1	203	104	110	108	94	77	56	70	4.3	7.6	1.11
**Stomach**	690 (8.74%)	2	41	31	47	70	91	104	103	201	3.6	7.8	2.03
**Esophagus**	436 (5.53%)	1	36	21	32	43	53	63	65	122	2.3	4.8	1.21
**Brain, central nervous system**	386 (4.89%)	47	148	33	33	31	28	23	18	25	2	2.9	0.39
Liver	379 (4.80%)	8	39	13	21	31	43	53	57	114	2	4.1	1.11
Lung	356 (4.51%)	2	57	23	30	37	42	45	43	77	1.9	3.7	0.82
Ovary	346 (4.38%)	4	73	37	45	50	45	38	27	27	1.8	3.2	0.43
Leukemia	327 (4.14%)	96	121	20	19	17	15	13	10	16	1.7	2.1	0.27
Colorectum	321 (4.07%)		35	16	19	27	36	43	49	96	1.7	3.5	‐
Lip, oral cavity	215 (2.72%)	3	33	18	22	26	28	28	23	34	1.1	2.1	0.38
Corpus uteri	185 (2.34%)		22	16	20	22	26	26	24	29	0.98	1.9	0.31
Non‐Hodgkin lymphoma	164 (2.08%)	16	56	14	14	14	13	12	9	16	0.87	1.3	0.21
Pancreas	136 (1.72%)		17	7	9	13	17	20	19	34	0.72	1.5	0.35
Kidney	112 (1.42%)	23	14	6	8	10	11	13	11	16	0.59	1	0.17
Gallbladder	70 (0.89%)		4	4	6	8	10	11	10	17	0.37	0.77	0.18
Thyroid	46 (0.58%)	2	5	1	3	5	6	7	7	10	0.24	0.48	0.09
Multiple myeloma	46 (0.58%)		11	6	2	6	4	5	4	8	0.24	0.43	0.08
Hodgkin lymphoma	40 (0.51%)	6	22	1	1	3	2	2	2	1	0.21	0.25	0.03
Bladder	39 (0.49%)	3	4	2	3	4	5	4	5	9	0.21	0.39	0.07
Larynx	37 (0.47%)		7	3	8	6	4	4	2	3	0.2	0.36	0.07
Melanoma of skin	31 (0.39%)		7	3	2	3	4	3	2	7	0.16	0.31	0.08
Oropharynx	27 (0.34%)	2	2	2	4	3	3	3	4	4	0.14	0.27	0.04
Nasopharynx	26 (0.33%)	1	13	2	2	1	1	2	2	2	0.14	0.2	0.03
Hypopharynx	26 (0.33%)		7	3	3	2	2	2	2	5	0.14	0.24	0.05
Vulva	24 (0.30%)			1	4	3	2	3	4	7	0.13	0.27	0.06
Vagina	15 (0.19%)		2	1	1	2	2	2	2	3	0.08	0.15	0.03
Salivary glands	16 (0.20%)		3	1	1	1	1	3	2	4	0.08	0.16	0.04
Kaposi sarcoma	6 (0.08%)		1			1	1	1	1	1	0.03	0.06	0.01
Mesothelioma	3 (0.04%)						1	1	1		0.02	0.04	0.01
**All cancers excluding non‐melanoma skin cancer**	7905 (100.18%)	340	340 (3.32%)	1422 (13.88%)	617 (6.02%)	740 (7.22%)	850 (8.30%)	900 (8.78%)	902 (8.80%)	809 (7.90%)	41.7	76.7	13.91

*Note*: *The incidence, prevalence proportions (prop.), crude rates, and ASR were reported per 100 000 people.

**FIGURE 1:**
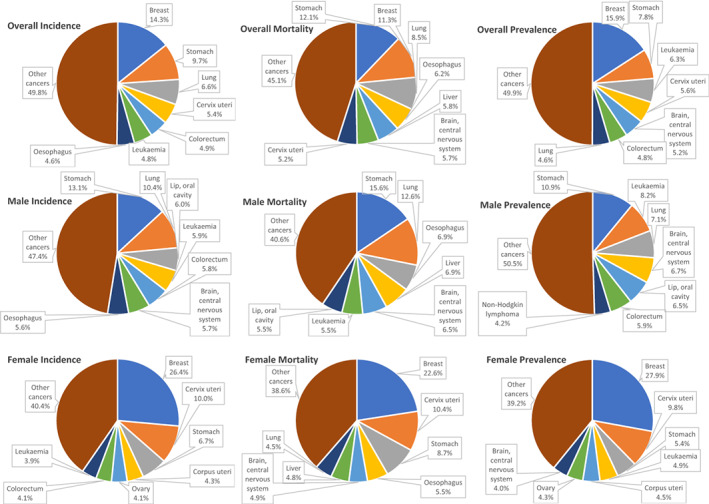
Top 5 cancers in Afghanistan, Incidence, Mortality, and 5‐year Prevalence.

Figure 1 illustrates the most prevalent and accountable for the most incidence and mortality in the Afghan population.

## 4 DISCUSSION

According to Global Cancer 2020 statistics for Afghanistan, the five most common cancers are breast, stomach, lung, cervix uteri, and colorectum. Cervical and colorectal cancers have replaced lip, oral cavity, and esophageal cancers on the list of the five most common cancers as of the 2018 WHO report.^4^ Similarly, the five most common cancers worldwide are similar, with breast, lung, colorectum, prostate, and stomach on the list.^15^ In addition to the GLOBOCAN report, which provides a global perspective, there is a paucity of comprehensive cancer studies specifically focused on Afghanistan. The limited number of regional and local studies conducted in Afghanistan,^5^, ^6^, ^7^, ^8^, ^16^, ^17^, ^18^, ^19^ emphasizes the critical need for further research in this context. Our study aims to address this gap by providing a detailed analysis of the prevalence, incidence, and mortality rates of cancer in Afghanistan. Moreover, we present the latest authentic statistics with epidemiological information on top cancers in Afghanistan, specifically Lung and Colorectum cancer, which have not been previously studied. By comparing our findings with existing studies and discussing the associated risk factors, our research offers a novel contribution to the understanding of cancer epidemiology in Afghanistan.

Afghanistan's inadequate cancer care infrastructure is a major issue due to a lack of data, skilled human resources, and policies and plans. Community awareness of cancer care is minimal, and few organizations have developed strategies to improve it.^18^ As a result, screening, prevention, and public education about cancer are critical in Afghanistan where patients must pay for the average cost of cancer treatment (nearly $600000) out of pocket, as there is no proper insurance coverage.^20^

Afghanistan's healthcare system is struggling to provide accessible cancer treatment to its population, with only one hospital in Kabul providing free mammography services to the country's 38.9 million residents.^21^ Poverty‐stricken areas lack medical facilities, making cancer a death sentence for many. Although breast cancer is the most common cancer among Afghan women, there is limited data on cancer incidence and mortality rates in the country.

The health insurance system in Afghanistan is underdeveloped, leaving only a small percentage of Afghans with access to health insurance. The National Cancer Control Program (NCCP) was established to improve cancer prevention, diagnosis, treatment, and palliative care services.^22^ The program aims to establish cancer registries across Afghanistan to collect data on cancer incidence and mortality rates. Despite limited opportunities for drug and radiotherapy treatments within Afghanistan, the NCCP has been able to establish cancer centers in Kabul, Herat, Mazar‐e‐Sharif, and Jalalabad.^23^

Unfortunately, there are limited opportunities for drug and radiotherapy treatments within Afghanistan. However, some international organizations provide support for cancer patients in the country. For example, the International Atomic Energy Agency (IAEA) provides training for Afghan doctors and nurses on radiation therapy.^24^ According to the International Agency for Research on Cancer (IARC), there were an estimated 20 000 cancer cases in Afghanistan in 2012, and this figure is expected to rise to almost 33 000 by 2030. Breast and CC account for half of all female cancer cases, killing over 2000 women each year.^24^

According to a report by the International Agency for Research on Cancer (IARC), Afghanistan has a lower incidence of cancer compared to other countries. The report states that in 2020, Afghanistan had an age‐standardized incidence rate of 108.8 per 100 000 people.^25^ There are several reasons why cancer incidence may be low in Afghanistan. One reason could be due to limited data availability and quality.^25^ Additionally, Afghanistan has a lower life expectancy compared to other countries,^26^ which could contribute to a lower incidence of cancer. However, it is important to note that cancer is still a significant health issue in Afghanistan.

### 4.1 Breast

Breast cancer (BC) is the most common, often diagnosed, and the leading cause of cancer mortality among the Afghan population, with an estimated 5‐year prevalence of 5930 cases, 3173 newly diagnosed cases, and 1783 deaths in 2020 (Figure 1). BC accounts for around 14.3% (12.5% globally) of all newly diagnosed cancer cases and 11.3% (6.9% globally) of all cancer deaths. It had roughly a 4.5‐fold higher incidence rate and a 2‐fold higher mortality rate in high‐ and very high‐HDI countries than in low‐ and medium‐HDI countries.^15^ BC incidence rates are highest in high‐income countries, while BC mortality rates are highest in low‐income countries due to inadequate screening and less efficient therapies.^27^, ^28^

Breast cancer incidence has continued to rise globally, most likely due to the adoption of increasingly Westernized lifestyles.^29^ The combination of multiple factors over several years is the cause of the majority of BCs. Some intrinsic risk factors include female sex, family history, race, genetic mutation, breast tissue density, and past radiation exposure.^30^ Obesity, alcohol consumption, smoking, lack of physical exercise, and radiation exposure, on the other hand, are modifiable and preventable risk factors that account for half or more incidences of BC.^31^, ^32^, ^33^ In addition, a case–control study found that age at menarche, age at first infant delivery, illiteracy, smoking, and a family history of cancer are significant risk factors for the development of BC in Afghan women.^18^

Screening/early detection and active preventive intervention are two main approaches to reducing BC's global burden.^34^ Screening methods include mammography, breast self‐examination (BSE), and clinical breast examination (CBE).^35^ Although mammography screening is also an effective tool, it is not cost‐effective in developing countries.^36^ Different factors, including demographic characteristics, awareness, literacy, and social and economic situations, might influence BC screening behaviors and should be considered when developing a cost‐effective approach to managing BC in Afghan women. The most viable option for Afghanistan seems to be the BSE, which is more culturally accepted and could be promoted effectively through awareness programs by international health organizations and the public health ministry.^17^, ^37^

BC mortality rates vary between countries and regions. In Afghanistan, the BC mortality rate among women is 22.6%, which is higher compared to neighboring countries such as Iran, Uzbekistan, China, and Turkmenistan, with mortality rates ranging from 14.8% to 20.7%. Pakistan and Tajikistan have higher mortality rates of 24.7% and 23.9%, respectively. The average BC mortality rate for Asia is 14.5%, while the world average is 15.6%.^15^ According to Table 4, BC incidence increases with age, and early detection through screening is crucial in reducing mortality rates. The CDC recommends mammograms every 2 years for women aged 50–74, reducing BC deaths by 26%.^38^, ^39^ However, Afghanistan does not have any national screening program, and projecting the potential impact of screening and early detection of BC mortality rates is not currently possible.

### 4.2 Stomach

Stomach or gastric cancer (GC) is Afghanistan's second‐most common, frequently diagnosed, and the leading cause of cancer mortality, with a 5‐year prevalence of 2913 cases, 2149 newly diagnosed cases, and 1918 deaths in 2020 (Figure 1). GC account for 9.7% (6% globally) of new cancer cases and 12.1% (7.8% globally) of cancer mortalities in Afghanistan.^15^ The GC rates are 2‐fold higher in men. In numerous South and Central Asian nations, including Iran, Afghanistan, Turkmenistan, and Kyrgyzstan, it is the most often diagnosed primary cause of cancer in men.^12^, ^40^

Risk factors, including Helicobacter pylori, tobacco, genetics, and diet, are known to be involved in causing GC.^33^, ^41^ Excessive salt consumption, a lack of fruits and vegetables, preserved foods, and red meat are all dietary factors.^42^ Several lifestyle factors have been associated with an increased risk of GC, including high salt intake, smoking, and low consumption of fruits and vegetables.^43^ A study by the American Association for Cancer Research confirmed that tobacco smoking moderately increases the risk of developing GC.^44^, ^45^ Adenocarcinomas comprise over 90% of gastric malignancies, which usually are caused due to chronic infection of Helicobacter pylori in the body of the stomach.^33^ Additionally, two studies in Kabul found a significant positive relationship between the inflammatory potential of the diet, dietary insulin index, and insulin load and the risk of GC in Afghan adults.^46^, ^47^

Endoscopy, the gold‐standard approach, has resulted in a considerable decline in national screening programs in Japan and South Korea.^48^ It is both expensive and invasive, making it an unfavorable option. Instead, genetic and molecular biomarkers are emerging as reliable and non‐invasive tools for detecting precancerous lesions and early stages of cancer.^49^ Despite the controversy, H. pylori eradication in the developing world remains a top priority in the fight against GC since it is serologically detectable.^50^ Diet and lifestyle changes are shown to be the most effective methods of preventing GC, especially in developed countries.^51^ In Afghanistan, urgent upper gastrointestinal endoscopy is recommended for individuals with dysphagia, those over 55 with weight loss, and specific symptoms, such as upper abdominal pain, reflux, or dyspepsia. However, there is no official guideline for endoscopy and its indications in Afghanistan. Further research is needed to improve screening and treatment options and to understand the higher incidence of GC in men.

This study shows that the GC's incidence is nearly double among men (13.1%) than women (6.7%), which follow the global trend. The age‐adjusted incidence rates of GC in men are approximately double those in women worldwide. Some research suggests this is because estrogen, a female hormone, helps protect the stomach from inflammation.^52^ Helicobacter pylori infection is closely associated with the development of GC, but the prevalence of infection does not substantially differ by sex.^53^ Although, some studies have shown a higher prevalence of H. pylori infection in males in certain regions.^54^

### 4.3 Lung

Lung cancer (LC) is the third most prevalent cancer diagnosed and the leading cause of cancer mortality in Afghanistan, accounting for 1.470 (6.6%) new cases and 1349 (8.5%) deaths in 2020. According to an estimate in 2020, almost 2.2 million new LC cases (11.4%) and 1.8 million cancer deaths (18.2%) occurred worldwide.^15^ LC is the leading worldwide cause of cancer morbidity and mortality in males, with men having about twice the rates as women.^55^ These rates are also three to four times higher in transitioned countries than in transitioning countries; however, this may change due to the tobacco epidemic since 80% of smokers aged 15 now live in LMICs.^15^ Western countries, including Denmark, the United States, and the United Kingdom, which have been linked with the tobacco epidemic since its inception and reached its peak in the middle of the last century, have reduced male LC mortality rates while increasing female LC mortality rates.^56^

Several risk factors with possible synergistic effects are associated with LC, including smoking, occupational and environmental risk factors, genetics, and gender, with smoking being the most significant factor in Afghanistan.^33^, ^57^, ^58^, ^59^ While cigarette smoking is the most common form of tobacco consumption, other tobacco products, such as water pipes and smokeless tobacco are becoming widely popular, and their consumption is even increasing among young adults, implying an increase in LC burden shortly.^60^ Unfortunately, tobacco products are widely available in Afghanistan, and smoking is socially accepted.^61^ Outdoor and occupational exposure to asbestos, radon, polycyclic aromatic hydrocarbons, and arsenic, and indoor air pollution from secondhand smoking, unventilated coal‐fueled stoves, and cooking gasses have all been related to LC, especially in Afghanistan.^62^, ^63^

Smoking cessation and reducing exposure to indoor pollution are important strategies in the prevention of LC.^64^ Extensive tobacco control programs have been effective in decreasing smoking rates, but since total abstinence programs have been considered a failure, combining abstinence programs with tobacco harm reduction programs can help lower smoking rates.^61^

Furthermore, implementing strict restrictions on outdoor air pollution and occupational exposure is crucial in Afghanistan. According to the findings of this study, women 15–39 is a higher incidence of LC than older ages. Indoor pollution from traditional stoves in low‐income countries is a significant concern. Studies from Bangladesh and China link exposure to cooking fumes with higher rates of LC in women, despite low smoking rates.^65^, ^66^ The WHO cautions that inhaling smoke from polluting household fuels and technologies harms health, especially for women and children engaged in cooking and firewood collection.^67^, ^68^ Moreover, young women worldwide have higher rates of LC than men, possibly due to different genetic risk factors such as abnormal genes related to cancer development or impaired DNA repair.^69^ Smoking is the leading cause of LC, but exposure to lung disease, occupational hazards, indoor air pollution, and drinking water with arsenic also increases the risk.^70^ To address this, public health policies should prioritize smoking cessation, tobacco control programs, and cleaner household fuels/technologies.

### 4.4 Cervix uteri

Cervix cancer (CC) is the fourth most prevalent diagnosed cancer in Afghanistan, with an estimated 5‐year prevalence of 2089 (5.6%) cases, 1200 newly diagnosed cases (5.4%), and 823 deaths (5.2%) occurring in 2020.^15^ CC is the most prevalent cancer diagnosed in 23 countries and the leading cause of cancer mortality in 36 nations, of which the great majority are in Sub‐Saharan Africa, Melanesia, South America, and South‐East Asia.^12^ It is more common in developing nations, most likely due to limited access to screening and the expensive cost of HPV vaccination.^71^

Cervix cancer is caused by the human papillomavirus (HPV), which is a necessary but not sufficient cause.^33^, ^71^ About 70% of all CC cases are caused by two high‐risk strains of HPV, 16 and 18.^71^ Some sexually transmitted infections (HIV and Chlamydia trachomatis), smoking, a larger number of childbirths, and long‐term use of oral contraceptives are also essential cofactors.^72^ CC is a largely preventable disease with a decreasing incidence thanks to improved screening and immunization against the most carcinogenic strains of HPV.^73^ Completing the prescribed immunization series, standardized screening, and education about contributory variables to urge avoidance of related risks are key preventative activities. Condom usage is around 70% effective in decreasing HPV transmission.^74^

The current screening method is Papanicolaou cytology (Pap) testing.^73^ An organized screening program used by developed nations frequently addresses crucial variables for efficient screening. Because of the shortage of healthcare resources in developing countries, they should implement a low‐cost/opportunistic screening program.^75^ Currently, most low‐income countries have ineffective BC screening and poorly documented coverage of CC opportunistic screening (less than 5%).^76^ In societies such as Afghanistan, where screening may not be acceptable, primary prevention through HPV vaccination is especially critical.^77^ A study in Kabul found that none of the women who developed CC had ever undergone cervical screening or heard of HPV vaccination.^78^ According to a cohort study, HPV vaccination in Afghanistan will be cost‐effective and beneficial, given the absence of a nationwide screening program for CC.^79^

The high incidence of CC among young women in Afghanistan (15–39 years) is a major concern due to the lack of national screening programs and HPV testing, which increases their risk. CC has had a devastating impact on women in Afghanistan, with 1200 new cases and 823 fatalities in 2020.^80^ The country ranks 117th in the world for age‐adjusted death rates from CC. Patient and healthcare delays are common, with 90.9% of women experiencing patient delay and 45.4% experiencing healthcare delay.^78^ Early diagnosis is crucial for successful treatment, but addressing cultural and societal factors that impact women's access to healthcare and screening programs is equally important. A multi‐pronged approach that includes medical interventions, education, and advocacy for increased access to healthcare services is necessary to improve screening and testing programs, increase awareness and education, and address cultural and societal factors. Failing to act will result in continued suffering and fatalities among women in Afghanistan, making it imperative to prioritize efforts to improve screening and testing programs and address the broader issues that impact women's access to healthcare.

### 4.5 Colorectum

Colorectal cancer (CRC) is the fifth most common cancer diagnosed in Afghanistan. An estimated 1804 new cases of colorectal cancer and 703 deaths occurred in Afghanistan in 2020. The CRC group consists of Colon, Rectum, and Anus cancers. Some studies indicate developed countries have four times more incidence rate, such as European regions, Australia/New Zealand, and Northern America have the highest incidence rates of colon cancer and Eastern Asia has the highest incidence of rectal cancer, whereas Africa and South‐Central Asia have the lowest rates.^12^ CRC is a sign of socioeconomic development, and incidence rates tend to climb consistently with increasing HDI in nations undergoing transition.^81^

Obesity, physical inactivity, high consumption of red or processed meat, tobacco smoking, and significant alcohol usage are all potentially modifiable risk factors.^33^ Therefore, losing body weight, remaining physically active, avoiding smoking, and limiting the intake of red meat and processed foods may assist in preventing this cancer.^82^ Because CRC is typically asymptomatic until warning symptoms develop in the late stages, implementing the screening program is critical to lowering cancer incidence and mortality rates.^83^ According to some studies, population‐based colorectal screening programs are usually not recommended in low‐incidence developing nations^84^; therefore, preventative and awareness campaigns are the most realistic choice.

A high incidence of CRC among young men aged 15–39 years old in Afghanistan has been reported in this study, which is consistent with previous research indicating a rising trend of CRC in young adults in developing countries.^85^ The underlying cause of this trend is not clear, but dietary factors may play a role. Specifically, a diet high in red or processed meats is associated with an increased risk of CRC due to the production of carcinogenic compounds during cooking and processing.^86^ Furthermore, a diet that is ultra‐processed, low in fiber, and high in added sugars has also been linked to a higher risk of CRC.^87^ Unfortunately, these dietary factors may be prevalent in many developing countries, including Afghanistan, where cultural and economic barriers can limit access to healthy foods. Afghanistan also has one of the world's highest malnutrition rates.

### 4.6 Recommendations for cancer prevention in Afghanistan

Cancer is becoming increasingly prevalent in Afghanistan, necessitating effective prevention and treatment strategies. To combat this, recommendations are proposed focusing on protection, early diagnosis, and education. Prevention efforts should include promoting lifestyle modifications, such as reducing tobacco use, maintaining a healthy diet, and increasing physical activity, alongside vaccination programs for hepatitis B and human papillomavirus. Early diagnosis is also critical to improving cancer outcomes and reducing mortality, and screening programs and diagnostic tests such as mammography, colonoscopy, and Pap tests should be established. Health education campaigns are essential to dispel myths and misunderstandings about cancer and its treatment, and policymakers should prioritize investing in healthcare infrastructure and resources, including cancer control programs and training healthcare professionals.

In light of recent political changes in Afghanistan, with the collapse of the republic government and the Taliban assuming control in August 2021, there is an urgent need to address the challenges faced by women, including their limited access to education and employment opportunities. In this context, it is crucial to initiate awareness campaigns regarding cervical and breast cancers, considering the low women's literacy rate of 30%, which further hinders their knowledge about these diseases. To ensure the dissemination of this awareness, information can be provided to husbands or individuals responsible for nuclear families. Furthermore, religious studies conducted in mosques and madrasas can serve as potential sources of information. Leveraging the power of social media, television, and radio, with a particular focus on radio due to its popularity in remote parts of Afghanistan, can prove instrumental. In these awareness efforts, the WHO should intensify its collaboration with the Ministry of Public Health (MoPH) in Afghanistan to maximize its impact. By implementing these measures, a more comprehensive approach can be achieved in promoting cancer awareness among Afghan women, thereby improving their health knowledge at this critical time.

Although the proposed recommendations can contribute to reducing the incidence and mortality of cancer in Afghanistan, a comprehensive approach that considers the country's health system and resources is necessary to implement them successfully. Further research is required to better understand the epidemiology of cancer in Afghanistan and develop region‐specific policies to prevent and control the disease. Urgent action is required to address the growing cancer burden in Afghanistan.

### 4.7 Limitation

GLOBOCAN collects cancer data using various methods depending on data availability, which affects the accuracy of its estimates. The validity and quantity of data vary from accurate counts to estimations based on sampling or nearby rates. A scoring system rates the quality and accuracy of the estimates, enabling users to evaluate country‐specific data. However, data quality and availability are improving over time owing to cancer incidence and mortality registry initiatives. Despite these limitations, the GLOBOCAN 2020 estimates are the most reliable cancer data and provide a credible basis for prioritizing cancer management globally.

## 5 CONCLUSION

Afghanistan is in an epidemiologic transition stage, and the burden of cancer is expected to rise due to population growth and aging, as well as a lack of adequate strategies to prevent, diagnose, and treat diseases, especially non‐communicable diseases, such as cancers. Analysis of the top five cancers, including breast, stomach, lung, cervix uteri, and colorectum, highlights the urgent need for improved healthcare infrastructure and comprehensive research in this context. Afghanistan faces significant challenges in terms of limited data availability, inadequate screening and prevention strategies, and lack of accessible and affordable cancer treatment options. This study underscores the critical importance of raising community awareness, developing effective screening programs, and implementing preventive interventions to address the burden of cancer in Afghanistan. Furthermore, the establishment of cancer registries, expansion of healthcare services, and collaboration with international organizations are vital steps toward improving cancer care and reducing mortality rates. Efforts should focus on addressing the risk factors specific to each cancer type, such as promoting breast self‐examination for breast cancer, implementing dietary and lifestyle interventions for stomach cancer, emphasizing smoking cessation and reducing indoor pollution for lung cancer, and emphasizing HPV vaccination and standardized screening for cervical cancer. By analyzing current policies and their implications, this study serves as a valuable resource for policymakers, healthcare providers, and international organizations working toward enhancing cancer prevention and control efforts in Afghanistan.

## AUTHOR CONTRIBUTIONS

**Nasar Ahmad Shayan:** Conceptualization (equal); data curation (equal); formal analysis (equal); methodology (equal); supervision (equal); validation (equal); writing – original draft (equal); writing – review and editing (equal). **Ali Rahimi:** Conceptualization (equal); data curation (lead); formal analysis (lead); methodology (lead); supervision (equal); validation (equal); visualization (lead); writing – original draft (lead); writing – review and editing (equal). **Hilal Özcebe:** Supervision (equal); validation (equal); writing – review and editing (lead).

## FUNDING INFORMATION

No funding was received for this study.

## CONFLICT OF INTEREST STATEMENT

The authors declare that they have no conflict of interest.

## ETHICS STATEMENT

Ethic statement for the article is not needed due to the nature of the review article.

## Data Availability

GLOBOCAN's Cancer Today data is free and publicly available (https://gco.iarc.fr/today/).
